# Personality and cognition: shoal size discrimination performance is related to boldness and sociability among ten freshwater fish species

**DOI:** 10.1007/s10071-024-01837-x

**Published:** 2024-03-02

**Authors:** Shi-Jian Fu, Na Zhang, Jie Fan

**Affiliations:** Laboratory of Evolutionary Physiology and Behavior, Chongqing Key Laboratory of Animal Biology, Chongqing Normal University, Chongqing, 401331 China

**Keywords:** Numerical ability, Interspecies, Coevolution, Personality, Cognition, Freshwater fishes

## Abstract

Several studies have reported that animals’ personalities are often correlated with individual differences in cognition. Here, we tested whether personality is related to cognition across species, focusing on 10 freshwater fishes and a task relevant for fitness, the ability to discriminate shoal size. Bolder species exhibited more ‘shuttle’ behavior for information sampling during shoal selection and showed high performance (HP) in the numerical discrimination than shyer species, i.e., low performance (LP) species. Species at both the high and low ends of sociability showed LP, possibly due to loosened selection pressure because of either no need to perform shoal size discrimination tasks frequently in nature for very high sociability species or decreased willingness and motivation to join and stay within shoals for very low sociability species. Notably, the numerical discrimination was sensitive to the numerical contrast ratio in LP species but not in HP species, suggesting that the numerical system used for size discrimination also varied between species. Overall, we demonstrated the interspecies relationship between personality and shoal size discrimination across fish species, suggesting an evolutionary link between numerical abilities and behavior.

**Supplementary Information:**

The online version contains supplementary material available at 10.1007/s10071-024-01837-x.

## Introduction

Animals, including fish, exhibit consistent differences in behavioral phenotypes (Biro and Stamps 2010). It has been found that behavioral traits such as boldness and sociability are often consistently different between individuals (i.e., personality traits) and often associated, forming suites of correlated behaviors named behavioral syndromes (Sih and Bell 2008; Wolf and Weissing 2012; Dubois and Binning 2022). Thus, individuals can be defined as having a proactive or reactive behavioral type accordingly, and similar differences can be observed at the interspecies level (Réale et al. 2010). It has been suggested that the existence of personality has substantial fitness consequences (Réale et al. 2010; Sih et al. 2012; Jolles et al. 2017). The ecological and evolutionary implications of personality in animals, including fish species, have drawn much attention from scientists in the last two decades (Sih and Bell 2008; Réale et al. 2010; Jolles et al. 2017; Tang and Fu 2019).

Recently, it has been suggested that personality might be related to cognition (Griffin et al. 2015; Lucon-Xiccato and Bisazza 2017). Cognition refers to how well animals acquire, process and handle information from the environment (Shettleworth 2010; Sih and Giudice 2012). It is often related to fitness because it is associated with, among the others, the ability to find food with good quality or quantity, successfully escape from predators, etc. (Giurfa 2019; Reichert et al. 2021; Liao et al. 2022). The link between personality and cognition may be due to the fact that proactive personalities (i.e., bold individuals) would obtain and process new information faster and hence might learn more quickly, and make decisions faster than reactive individuals (Griffin et al. 2015; Griffin and Guez 2014). This has been demonstrated in fish species such as mormyrid fish (*Gnathonemus petersii*), zebrafish (*Danio rerio*) and brook trout (*Salvelinus fontinalis*) (White et al. 2017; Kareklas et al. 2017; Daniel and Bhat 2020, 2023), as well as in reptiles (Carazo et al. 2014), birds (Ferreira et al. 2019) and mammals (Schuster et al. 2017). However, all these previous studies were focused only at the inter-individual level. Investigation of whether similar relationships manifest at the interspecies level may elucidate the adaptation and evolution of cognition and personality and the coexistence between different personality and cognition types in light of fitness consequences in nature (Lucon-Xiccato and Dadda 2017a).

The present study aimed to test the relationship between personality and numerical cognition in freshwater fish species at an interspecies level. Numerical abilities are widespread in animal kingdoms, including fish species (Bisazza et al. 2014; Agrillo and Bisazza 2018; Lucon-Xiccato and Dadda 2017a, b; Bisazza and Gatto 2021; Bisazza and Santacà 2022). Two common methods have been frequently used in numerical ability tests, e.g., operant conditioning with artificial stimuli training (Bisazza and Santacà 2022) or spontaneous shoal preference (Lucon-Xiccato and Bisazza 2017). The latter is widely used in collective-living fish species because it is a cognitive task performed frequently in their natural environment (Thorn et al. 2014; Lucon-Xiccato and Bisazza 2017). This is because fish species evolve to prefer to stay in shoals with more members as protection from predation due to the so-called ‘dilution effect’ (Foster and Treherne 1981), ‘confusion effect’ (Landeau and Terborgh 1986) and ‘many eye-effect’ (Pulliam 1973). We anticipated that bolder fish species might have superior numerical ability, as they obtain and process numerical information faster than shyer species. According to the relationship between sociability and shoal size discrimination performance, a previous study in guppies (*Poecilia reticulata*) found that sociability was negatively correlated with numerical ability (Lucon-Xiccato and Dadda 2017a). The authors suggested that this might be because fish with low sociability likely spend more time alone, and are required to solve the problem of choosing between different shoals more often (Cote et al. 2012; Lucon-Xiccato and Dadda 2017a). However, this might also be because at the intraspecific level, individuals with high sociability generally have lower boldness (Réale et al. 2010) and hence show poor shoal size discrimination ability. At the interspecies level, the relationship might be different, or even opposite, as high sociability has often been associated with enhanced cognitive abilities at the interspecific level (Dunbar and Shultz 2007). Furthermore, the relationship might be strengthened by the fact that fish with high sociability would be more motivated to join the large shoal (Irving and Brown 2013; Trompf and Brown 2014).

To fulfill our goal, we selected nine cyprinid fish species from Cypriniformes and one cichlid from Persiformes. All experimental fish are collective-living and docile species and most easily available. We measured the personality traits of boldness and sociability, numerical ability by a spontaneous shoal preference test, and behavioral traits during shoal size discrimination (Bai et al. 2019). We anticipated that shoal discrimination performance would be positively correlated with boldness at the interspecies level, whereas the possible relationship or the direction of correlation between sociability and numerical ability remained to be tested.

## Materials and methods

### Experimental fish and maintenance

**Fig. 1:**
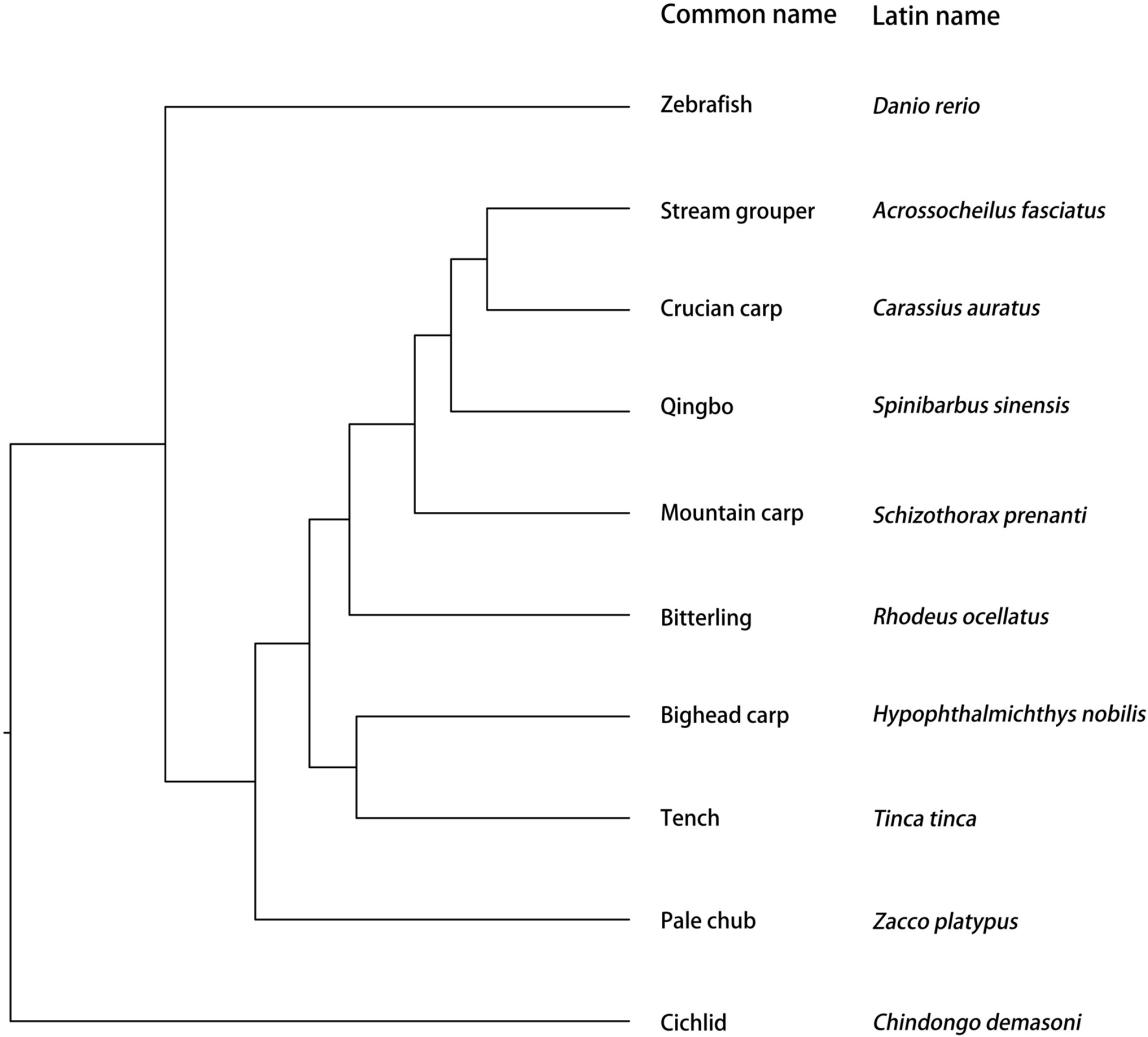
The phylogenetic relationship of the experimental fishes used in the present study

Experimental fish included 10 fish species, i.e., cichlid (*Labidochromis caeruleus*), zebrafish (*Danio rerio*), mountain carp (*Schizothorax prenanti*), pale chub (*Zacco platypus*), tench (*Tinca tinca*), bitterling (*Rhodeus ocellatus*), qingbo (*Spinibarbus sinensis*), stream grouper (*Acrossocheilus fasciatus*), crucian carp (*Carassius auratus*) and bighead carp (*Hypophthalmichthys nobilis*). All species are cyprinid species from Cypriniformes, except the cichlid that is from Perciformes (Fig. 1). Fish were obtained from two places, i.e., a local fish hatchery (Yongchuan, Chongqing, China) or the Mashi Aquarium (Shapingba, Chongqing, China). The experimental fish were juveniles with similar age except for zebrafish (*Danio rerio*), and only female zebrafish were used in this experiment. The experimental fish were used only once for measurement of personality and shoal size discrimination. The sample sizes and body sizes are listed in Table 2 and Table 3. The sample size of stream grouper was much less than other species because it was relatively difficult to obtain. Fish were reared in the indoor circulating rearing system described in a previous study (Xiong et al. 2018) for two weeks of acclimation before any experimental measurement. During the acclimation period, the fish were fed to apparent satiation daily with commercial feed at 9:00 AM. The water temperature was maintained at 25.0 ± 0.5 °C. All fish were fasted for 24 h before any measurements.

### Apparatus for shoal preference measurement

The experimental arena for the measurement of numerical discrimination ability has been previously described (Bai et al. 2019). Briefly, a tank was constructed from transparent polymethyl methacrylate (70 × 35 × 35 cm) (Supplemental Fig. 2a). The tank was divided into a test compartment in the middle (50 × 35 × 35 cm) and two stimulus compartments (10 × 35 × 35 cm) on the right- and left-hand sides by transparent glass partitions. The arena was illuminated by a 15-W LED light at each end. A webcam (Logitech Pro 9000; Logitech Company, Suzhou, China) connected to a remote computer was placed directly over the test tank, which was used to record the positions of the test fish during the tests.

**Fig. 2:**
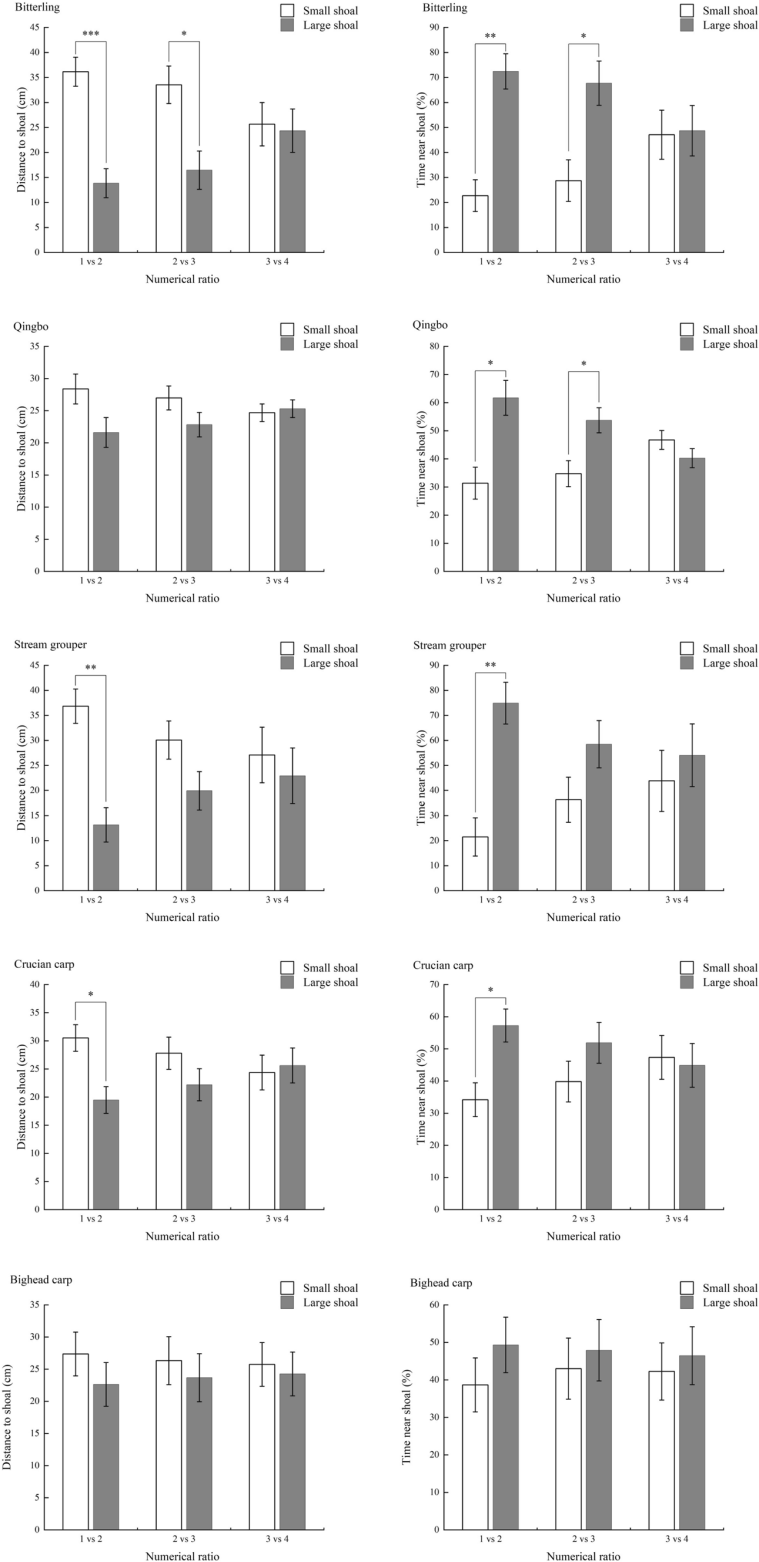

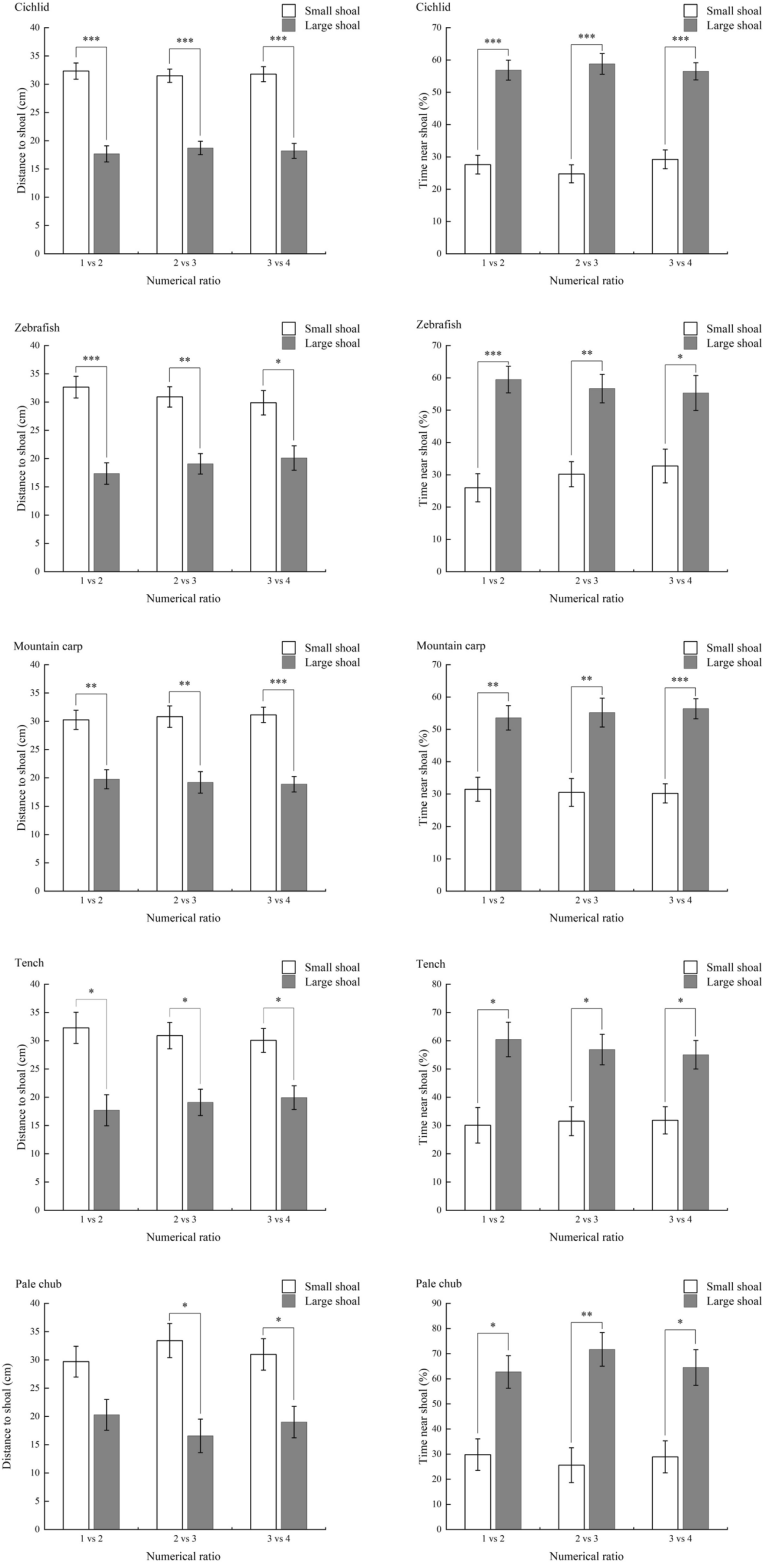
The distance to either small- (empty column) or large-size (gray column) shoals and percentage time near either shoal of ten experimental fish species under different numerical contrast ratios (mean ± S.E., N varied from 10 to 12 in steam grouper and varied from 20 to 24 in other species; see more detail in Tables 2 and 3 for sample size and body size) *** *P* < 0.001, ** *P* < 0.01, * *P* < 0.05

### Apparatus for personality measurement

The arena for the sociability measurement was the same as that for the shoal preference but with different test (40 × 35 × 35 cm) and stimulus (15 × 35 × 35 cm) compartments (Supplemental Fig. 2b). The arena for the boldness measurement was also a rectangular tank made of polymethyl methacrylate (70 × 35 × 35 cm). However, it was divided into an open area (55 × 35 × 35 cm) and shelter area (15 × 35 × 35 cm) by an opaque partition (Supplemental Fig. 2c) (see details in Liu and Fu 2017). A portable door was adjacent to the partition. Plastic plants and gravel pebble stones were placed in the shelter area. Similarly, a webcam connected to a remote computer was used to record the positions of the test fish during the tests.

### Procedure for shoal preference measurement

The arena was filled with a depth of 10 cm water with a temperature of 25 °C for all measurements. The numerical discrimination ability of the experimental species was measured under three numerical contrast ratios, i.e., 1 *vs*. 2, 2 *vs*. 3 and 3 *vs*. 4. The procedure has been previously described (Xiong et al. 2018; Bai et al. 2019). In brief, stimulus fish with different numbers were introduced to the stimulus area and allowed to recover for 10 min. Then, test fish were transferred from the rearing tank to the test area with water in a plastic cylinder and allowed to recover in the device for 2 min. Then, the holding device was gently removed, and the position of the test fish was recorded for 20 min at 15 frames s^−1^ by the webcam. The sample sizes (i.e. repetition) varied from 10 to 12 in each contrast ratio in one fish species (i.e. stream grouper) and varied from 20 to 24 for the remaining fish species (see Table 2 for more detail).

The cognitive task of shoal size discrimination depends on how fish sample and process information and make decisions (Sih and Del Giudice 2012; Lucon-Xiccato et al. 2017). Thus, if personality is related to the numerical ability across species, we anticipate that the behavior pattern might also vary across species during their information collection and handling processes when conducting the shoal preference task. Benefitting from recent developments in animal trajectory capture software (e.g., idTracker, Pérez-Escudero et al. 2014), we can now easily acquire the trajectory of fish and calculate the movement traits during spontaneous shoal preference activities. Thus, in the present study, the position of the test fish was analyzed by the automated tracking software program idTracker (v 2.1) (Pérez-Escudero et al. 2014). This program automatically tracked the centroid of each fish in each trial and provided the x and y coordinates of each fish in each video.

It was assumed that a test fish had selected a shoal once they were within the 15 cm (approximately 3 body lengths) area adjacent to the partition facing the stimulus tank (i.e., within the preference zone). Preference, i.e., percentage of time spent in each preference zone, and distance to the partition facing either small or large stimulus shoals was computed by the coordinates.

To describe the characteristics of the swimming behavior of the test fish during the shoal preference test, we selected four variables, i.e., shuttling frequency of the test fish across the stimulus shoal, percentage of time spent in the neutral area, median speed and percentage of time spent moving. The shuttling behavior was artificially observed (Bai et al. 2019), and the time in the neutral area was computed by the coordinates. The swimming speed was also calculated by the coordinates as described previously, whereas time spent moving was the proportion of swimming speed greater than 1.75 cm s^−1^ (Miller and Gerlai 2008). We used the mean values of all three numerical contrasts of each species for data analysis.

### Procedure for personality measurement

The process of sociability measurement has been described previously (Killen et al. 2016). Stimulus shoals consisting of six individuals were transferred to either the right or left stimulus area alternatively (to present side preference), whereas another stimulus area remained empty. After 10 min, one test fish was transferred to the neutral area, and the process was the same as that in the shoal preference test. The movement of focal fish was filmed for 20 min. The position of the test fish was analyzed by idTracker (v 2.1). Sociability was estimated by measuring the distance of the test fish to the stimulus shoal and the percentage of time the test fish spent within the preference zone, i.e., 15 cm of each stimulus shoal area (Miller and Gerlai 2008; Xiong et al. 2018).

The process of boldness measurement has also been described previously (Tang and Fu 2019). Briefly, one test fish was transferred to the shelter area and allowed to recover for 30 min. Then, the door between the open and shelter areas was lifted gently and the movement of the test fish was filmed by a webcam for 30 min. Boldness was estimated by measuring the latency (when the test fish first emerged from shelter area to open area) and frequency of test fish entering the open area (the total number of test fish entering the open area divided by the observation period), which was calculated from videos artificially (Tang and Fu 2019). The use of single emergence test assay might have a potential weakness as some individuals are more stress than others in a novel environment (Beckmann and Biro 2013). In the present study, we used single trial because previous studies found that both boldness and sociability traits were quite repeatable in cyprinid fish species (e.g. Tang and Fu 2019). Furthermore, the present study measured the personality traits at interspecific level difference rather than inter-individual level difference, and all selected fish were docile species.

### Data analysis

SPSS was used to analyze the data. In the shoal preference test, the difference of time near shoal and distance to shoal was tested by a linear mixed model, using ratio and shoal size as the main effect, fish ID as a random factor and body mass as a covariate. Whether fish species could distinguish any given numerical ratio was judged by the significant difference in distance to large and small stimulus shoals and percentage time near the large and small stimulus shoals. The fish species were classified as high performance (HP) of numerical discrimination ability if they can distinguish all numerical ratios, whereas the remaining species were classified as low performance (LP) of numerical discrimination ability species. The species swimming traits during shoal preference measurement (mean values of three numerical contrasts of each species were used for data analysis) and variables of personality measure was tested by one-way analysis of covariance, using body mass as covariate. The difference between species was further compared by Duncan multiple comparison. The relationship between personality traits and other variables among species was tested by spearman correlation. The difference of variables between HP species and LP of numerical discrimination ability was tested by *t* test. The spontaneous movement traits and personality of fish species with HP and LP were described as boxplots. The remaining data were described as the mean ± S.E., and *P* < 0.05 was used as the threshold for statistical significance.

## Results

### Shoal preference performance

The species showed large interspecific differences in numerical ability. Five fish species, i.e., cichlid, zebrafish, mountain carp, tench and pale chub, could distinguish all numerical ratios, as there was a significant difference in distance to large and small shoal sizes and percentage time near the stimulus shoal under all three numerical contrast ratios except the distance of the pale chub under the 1 *vs*. 2 condition (Fig. 2; Table 1). Furthermore, the numerical contrast ratio showed no significant effect on either variable in the abovementioned HP fish species, i.e., the performance was ratio insensitive.

**Table 1:** The effect of stimulus shoal size and numerical ratio on the shoal size discrimination performance of experimental fish species based on a linear mixed model analysis (with the fish ID as a random variable and body mass as a covariate)

	Variable	Ratio effect	Size effect	Interaction effect
Cichlid	Distance to shoal	*F*_2,122_ = 0.004	*F*_1,122_ = 155.8	*F*_2,122_ = 0.257
		*P* = 0.996	*P* < 0.001*	*P* = 0.773
	Time near shoal	*F*_2,122_ = 0.065	*F*_1,122_ = 155.8	*F*_2,122_ = 0.656
		*P* = 0.937	*P* < 0.001*	*P* = 0.521
Zebrafish	Distance to shoal	*F*_2,134_ = 0.000	*F*_1,134_ = 58.92	*F*_2,134_ = 1.107
		*P* = 1.000	*P* < 0.001*	*P* = 0.365
	Time near shoal	*F*_2,134_ = 0.040	*F*_1,134_ = 53.77	*F*_2,134_ = 0.727
		*P* = 0.961	*P* < 0.001*	*P* = 0.485
Mountain carp	Distance to shoal	*F*_2,132_ = 0.000	*F*_1,132_ = 51.82	*F*_2,132_ = 0.843
		*P* = 1.000	*P* < 0.001*	*P* = 0.433
	Time near shoal	*F*_2,132_ = 0.020	*F*_1,132_ = 45.54	*F*_2,132_ = 0.866
		*P* = 0.980	*P* < 0.001*	*P* = 0.423
Tench	Distance to shoal	*F*_2,134_ = 0.000	*F*_1,134_ = 38.73	*F*_2,134_ = 0.429
		*P* = 1.000	*P* < 0.001*	*P* = 0.652
	Time near shoal	*F*_2,134_ = 0.056	*F*_1,134_ = 34.91	*F*_2,134_ = 0.223
		*P* = 0.946	*P* < 0.001*	*P* = 0.801
Pale chub	Distance to shoal	*F*_2,136_ = 0.000	*F*_1,136_ = 30.16	*F*_2,136_ = 0.878
		*P* = 1.000	*P* < 0.001*	*P* = 0.418
	Time near shoal	*F*_2,136_ = 0.072	*F*_1,136_ = 49.30	*F*_2,136_ = 0.542
		*P* = 0.930	*P* < 0.001*	*P* = 0.583
Bitterling	Distance to shoal	*F*_2,130_ = 0.000	*F*_1,130_ = 20.26	*F*_2,130_ = 4.267
		*P* = 1.000	*P* < 0.001*	*P* = 0.016*
	Time near shoal	*F*_2,130_ = 0.003	*F*_1,130_ = 19.07	*F*_2,130_ = 4.346
		*P* = 0.997	*P* < 0.001*	*P* = 0.015*
Qingo	Distance to shoal	*F*_2,136_ = 0.000	*F*_1,136_ = 5.370	*F*_2,136_ = 2.017
		*P* = 1.000	*P* = 0.022*	*P* = 0.137
	Time near shoal	*F*_2,136_ = 0.239	*F*_1,136_ = 23.65	*F*_2,136_ = 2.882
		*P* = 0.788	*P* < 0.001*	*P* = 0.061
Stream grouper	Distance to shoal	*F*_2,62_ = 0.000	*F*_1,62_ = 11.82	*F*_2,62_ = 2.045
		*P* = 1.000	*P* = 0.001*	*P* = 0.104
	Time near shoal	*F*_2,62_ = 0.012	*F*_1,62_ = 11.52	*F*_2,62_ = 2.220
		*P* = 0.908	*P* = 0.001*	*P* = 0.117
Crucian carp	Distance to shoal	*F*_2,136_ = 0.000	*F*_1,136_ = 5.086	*F*_2,136_ = 2.421
		*P* = 1.000	*P* = 0.026*	*P* = 0.093
	Time near shoal	*F*_2,136_ = 0.002	*F*_1,136_ = 4.727	*F*_2,136_ = 2.177
		*P* = 0.998	*P* = 0.031*	*P* = 0.117
Bighead carp	Distance to shoal	*F*_2,134_ = 0.000	*F*_1,134_ = 1.056	*F*_2,134_ = 1.056
		*P* = 1.000	*P* = 0.306	*P* = 0.898
	Time near shoal	*F*_2,134_ = 0.020	*F*_1,134_ = 1.086	*F*_2,134_ = 0.103
		*P* = 0.981	*P* = 0.299	*P* = 0.902

^*^Significant at *P* < 0.05

Neither bitterling nor qingbo could distinguish 3 *vs*. 4 numerical contrast ratios, whereas stream grouper and crucian carp could only distinguish 1 *vs*. 2 numerical contrast ratios, as indicated by significant difference in either distance to, or percentage time near, the large and small stimulus shoals. Furthermore, there was no significant difference under all three numerical contrast ratios in bighead carp, although fish showed a higher time and shorter distance of values to large shoals. Notably, the difference in both variables between large and small shoal sizes decreased with numeral ratios in all five abovementioned LP species, i.e., the performance was ratio dependent.

### Spontaneous movement trait during the shoal size discrimination task

**Fig. 3:**
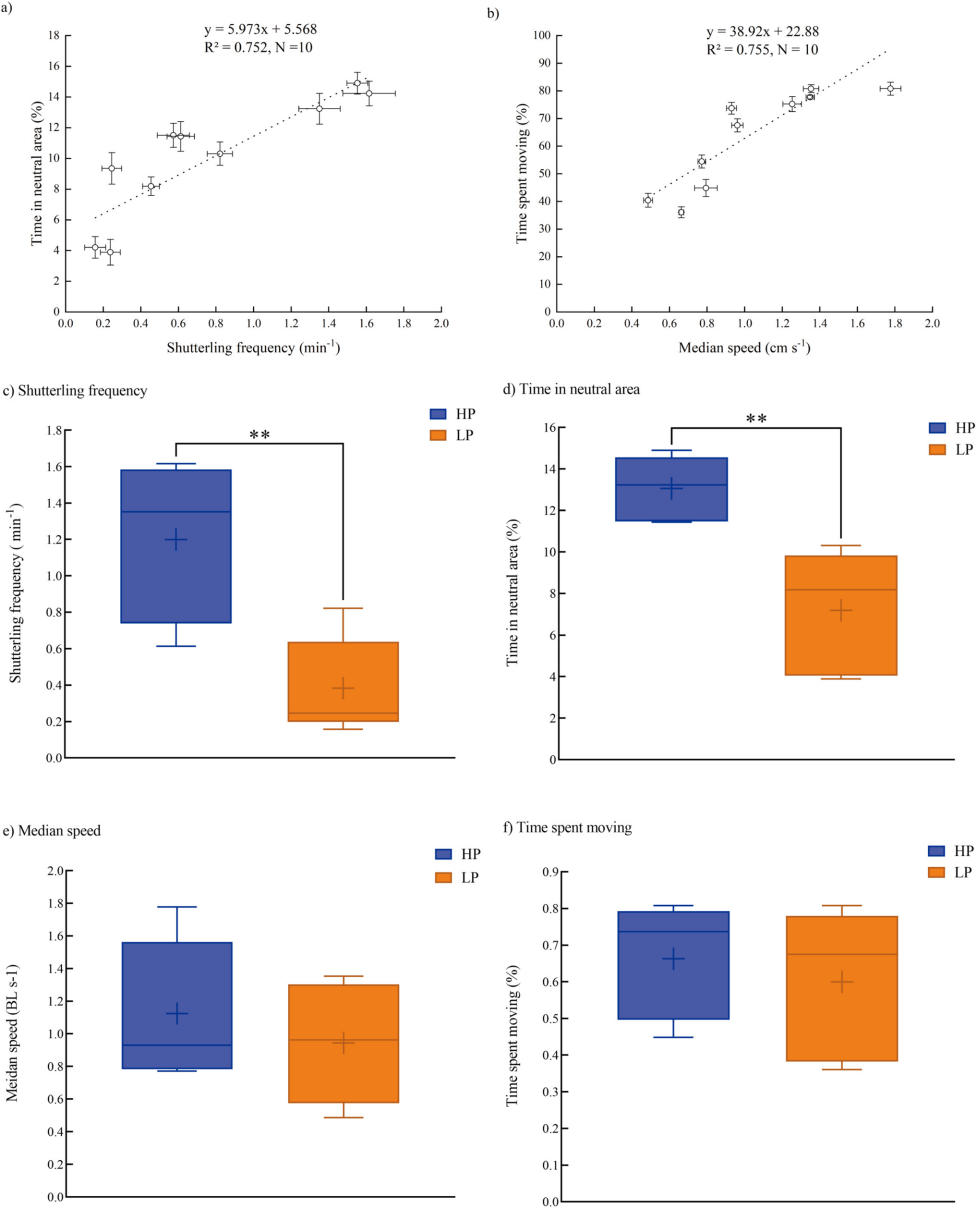
The relationship between movement traits during the shoal size discrimination task among ten experimental fish species, and boxplots of spontaneous movement traits between fish species with high (HP) and low (LP) shoal size discrimination performance *** *P* < 0.001, ** *P* < 0.01, * *P* < 0.05

All variables varied significantly among species (Table 2; Supplemental Table 1). At the interspecific level, the shuttling frequency was positively correlated with the percentage of time spent in the neutral area, whereas the time spent moving was positively correlated with the median speed (Fig. 3a, b). However, there was no significant correlation between these four variables except for the abovementioned two correlations (Supplemental Table 2).

**Table 2:** Traits of the movement behavior of fish species during the shoal size discrimination task (mean ± S.E.)

Species	*N*	Body mass (g)	Body length (cm)	Shuttering frequency (min^−1^)	Time in neutral area (%)	Time spent moving (%)	Median speed (BLs^−1^)
Cichlid	64	1.66 ± 0.04	4.21 ± 0.03	1.55 ± 0.06^a^	14.90 ± 0.71^a^	77.71 ± 0.604^a^	1.35 ± 0.02^b^
Zebrafish	70	0.76 ± 0.02	3.33 ± 0.02	1.35 ± 0.11^a^	13.23 ± 1.00^ab^	80.78 ± 2.38^a^	1.78 ± 0.06^a^
Mountain carp	69	1.33 ± 0.03	4.56 ± 0.04	1.62 ± 0.14^a^	14.23 ± 0.80^a^	73.68 ± 2.12^ab^	0.93 ± 0.03^c^
Pale chub	72	1.17 ± 0.04	4.42 ± 0.05	0.87 ± 0.13^b^	11.50 ± 0.78^bc^	44.85 ± 3.08^d^	0.79 ± 0.06^d^
Tench	70	1.84 ± 0.04	4.65 ± 0.04	0.61 ± 0.07^bc^	11.43 ± 0.97^bc^	54.42 ± 2.35^c^	0.77 ± 0.02^de^
Bitterling	68	2.63 ± 0.05	4.86 ± 0.03	0.16 ± 0.06^e^	4.20 ± 0.70^e^	75.20 ± 2.71^a^	1.25 ± 0.05^b^
Qingbo	72	2.00 ± 0.06	4.88 ± 0.04	0.82 ± 0.07^b^	10.30 ± 0.76^ cd^	40.40 ± 2.51^de^	0.49 ± 0.02^f^
Stream grouper	34	1.05 ± 0.03	3.91 ± 0.04	0.24 ± 0.05^de^	3.89 ± 0.84^e^	67.52 ± 5.36^b^	0.96 ± 0.03^c^
Crucian carp	71	2.12 ± 0.04	4.63 ± 0.03	0.45 ± 0.04^ cd^	8.19 ± 0.60^d^	80.77 ± 1.43^a^	1.35 ± 0.04^b^
Bighead carp	70	1.06 ± 0.03	4.07 ± 0.04	0.25 ± 0.05^de^	9.35 ± 0.80^ cd^	36.03 ± 1.97^e^	0.66 ± 0.01^e^

a, b, c, d, e, f, superscript without a common letter are significantly different (*P* < 0.05)

The five HP species showed significantly higher shuttling frequency and time in the neutral area than the LP species, whereas there was no significant difference in the other two variables (Fig. 3c, d, e, f).

### Personality

**Fig. 4:**
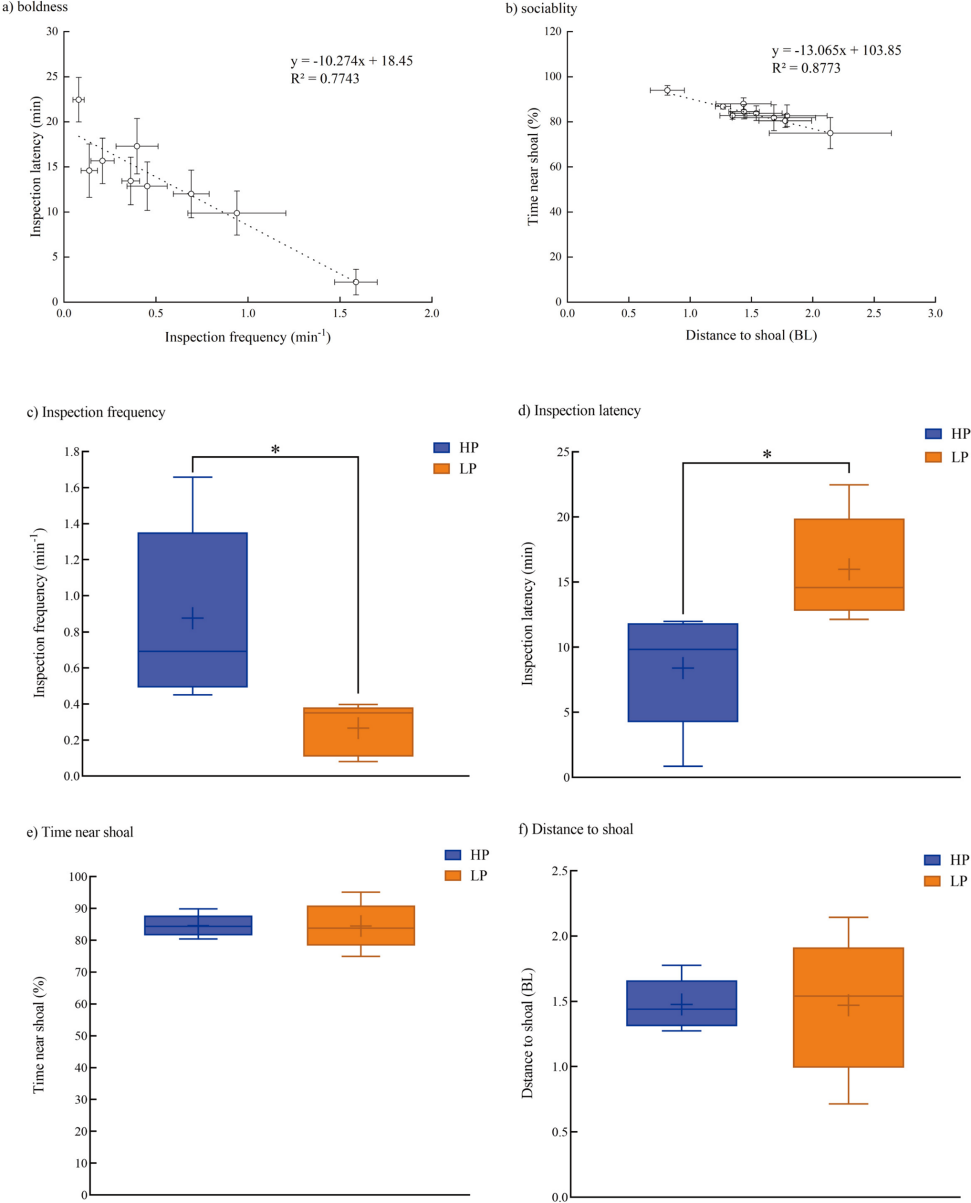
The relationship between variables of sociability or boldness among ten experimental fish species, and boxplots of personality between fish species with high (HP) and low (LP) scores among ten experimental fish species *** *P* < 0.001, ** *P* < 0.01, * *P* < 0.05

All four personality variables varied significantly between species (Table 3, supplemental Table 1). Two boldness variables, i.e., inspection latency, were negatively correlated with inspection frequency at the interspecies level, whereas two sociability variables, i.e., time near the shoal, were negatively correlated with distance to the shoal at the interspecies level (Fig. 4a, b). HP species showed shorter latency but a higher frequency of inspection than LP species (Fig. 4c, d). However, there was no significant difference in sociability variables between HP and LP species (Fig. 4e, f).

**Table 3:** Personality traits of ten experimental fish species (mean ± S.E., *N* = 20)

Species	Body mass (g)	Body length (cm)	Boldness		Sociability	
			Inspection frequency (min^−1^)	Inspection latency (min)	Time near shoal (%)	Distance to shoal (BLs^1^)
Cichlid	1.97 ± 0.07	4.34 ± 0.05	1.66 ± 0.09^a^	0.86 ± 0.36^d^	82.70 ± 1.61^bc^	1.35 ± 0.10^bc^
Zebrafish	0.76 ± 0.04	3.34 ± 0.05	1.04 ± 0.27^b^	7.64 ± 2.00^ cd^	89.89 ± 1.68^ab^	1.27 ± 0.14^bc^
Mountain carp	1.30 ± 0.06	4.48 ± 0.07	0.53 ± 0.11^c^	9.83 ± 2.31^bc^	85.73 ± 3.55^abc^	1.56 ± 0.23^ab^
Pale chub	3.16 ± 0.12	6.31 ± 0.24	0.69 ± 0.10^c^	11.99 ± 2.65^bc^	84.43 ± 3.20^abc^	1.44 ± 0.12^abc^
Tench	1.92 ± 0.06	4.53 ± 0.05	0.45 ± 0.08^cd^	11.68 ± 2.42 ^bc^	80.41 ± 2.92^bc^	1.78 ± 0.21^ab^
Bitterling	1.97 ± 0.07	4.62 ± 0.05	0.40 ± 0.11^cd^	17.29 ± 3.07^b^	74.94 ± 6.92^c^	2.14 ± 0.50^a^
Qingbo	2.63 ± 0.07	6.58 ± 0.06	0.36 ± 0.05^cd^	13.43 ± 2.63^bc^	86.77 ± 1.11^abc^	1.27 ± 0.06^bc^
Stream grouper	1.70 ± 0.09	4.81 ± 0.09	0.35 ± 0.08^cd^	12.13 ± 3.14^bc^	83.80 ± 3.27^abc^	1.54 ± 0.21^ab^
Crucian carp	2.18 ± 0.09	4.57 ± 0.08	0.08 ± 0.03^e^	22.45 ± 2.47^a^	95.11 ± 1.76^a^	0.71 ± 0.08^c^
Bighead carp	1.29 ± 0.04	4.43 ± 0.05	0.14 ± 0.04^de^	14.58 ± 2.95^bc^	81.82 ± 5.73^bc^	1.68 ± 0.34^ab^

a, b, c, d, e, superscript without a common letter are significantly different (*P* < 0.05)

### Correlation between movement traits during the shoal preference test and personality

**Fig. 5:**
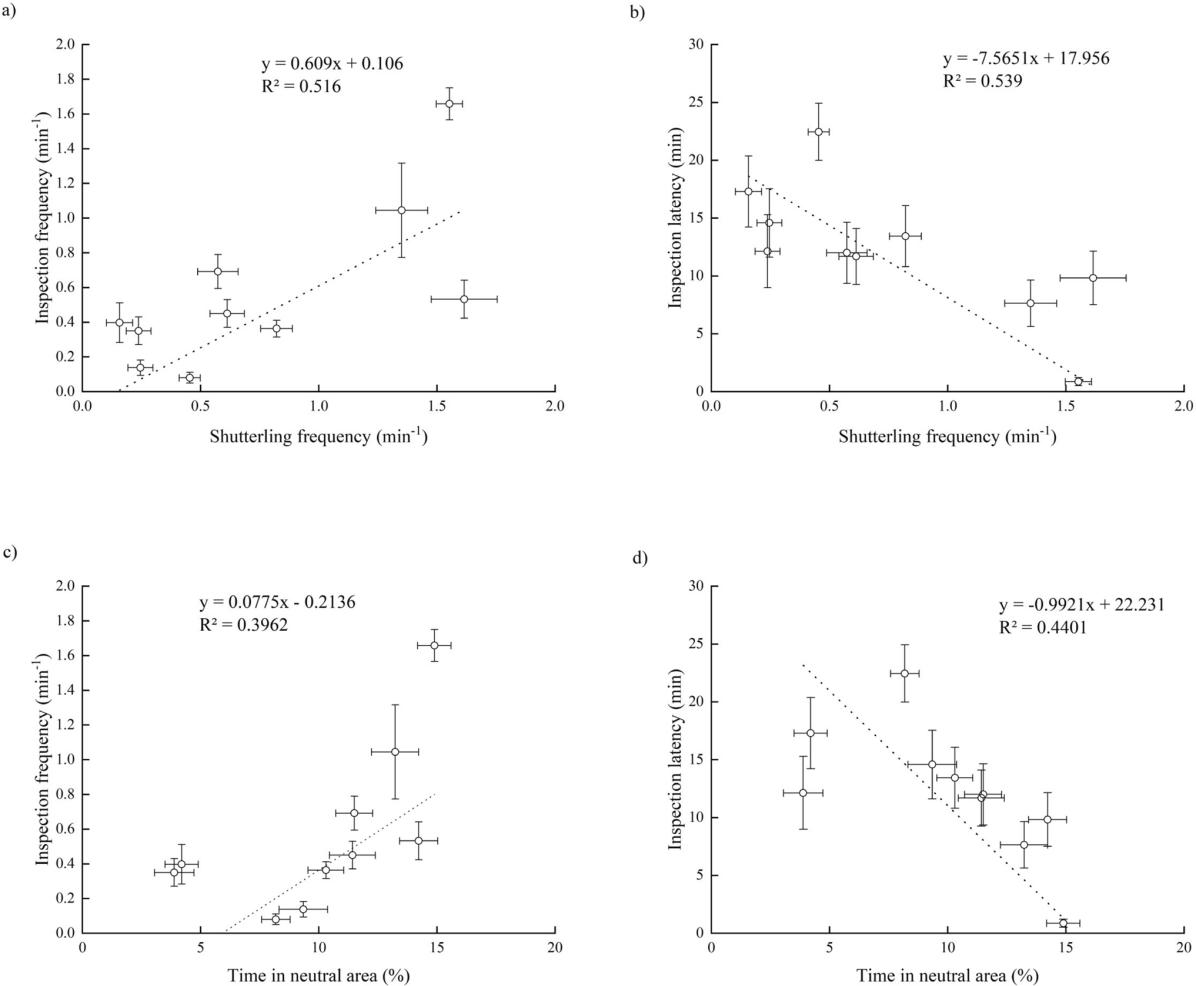
The relationship between movement traits during the shoal size discrimination task and personality among ten experimental fish species

Median speed and time spent moving showed no significant relationship with any of the personality variables (Supplemental Table 2). However, both shuttling frequency and percentage time spent in the neutral area were positively correlated with inspection frequency and negatively correlated with inspection latency among the 10 species (Fig. 5a, b, c, d).

## Discussion

### The relationship between boldness and shoal size discrimination ability

The relationship between personality and cognition has been previously proven at the intraspecific level (Griffin et al. 2015; Lucon-Xiccato and Dadda 2017a; Daniel and Bhat 2023). It has been suggested that the willingness and efficiency of animals to sample environmental information might lie under the positive correlation between boldness and cognition (White et al. 2017; Kareklas et al. 2017; Daniel and Bhat 2023). In the present study, we found a positive relationship between boldness and shoal size discrimination performance across 10 selected freshwater fish species. This suggests that the driving force of such coevolution between personality and cognition might also work at the interspecific level. Moreover, the interconnection between boldness and cognition is reflected by spontaneous shoal preference behavior: (1) all the HP species showed higher shuttling frequency and spent more time in the neutral area than the LP species, and (2) both shuttling frequency and time spent in the neutral area were positively correlated with two boldness variables among 10 species. In short, the present study suggests that species with high boldness sample information more frequently during the shoal size discrimination task, thus exhibiting superior numerical ability as they join the large shoal with more acuity. However, although shoal preference is a frequently performed cognitive task with high ecological relevance in the field for collective-living fish species (Landeau and Terborgh 1986; Krause 2002; Lucon-Xiccato and Dadda 2017a), the experiment results based on shoal preference is not able to clearly differentiate the interpretation between numerical ability and social preference. It is all possible that no preference in LP fish species when measured at high numerical contrast (e.g., 3 *vs*. 4) is due to lower benefits of choosing the larger of two groups when overall group size is high rather than cognitive limitations (Wright et al. 2023). Further investigation using operant conditioning with artificial stimuli training would be necessary for supporting the conclusion about the relationship between personality and numerical ability at interspecific level made in the present study.

### The relationship between sociability and shoal size discrimination ability

Sociability has long been considered a personality trait that could be associated with the shoal size discrimination ability in fish species (Magurran and Seghers 1991; Irving and Brown 2013; Cattelan et al. 2017), as individuals with high sociability might be more rushed to find a shoal and thus would have superior numerical ability during shoal preference (Irving and Brown 2013; Trompf and Brown 2014). The association might be strengthened among species, as at the interspecific level, sociability has been associated with enhanced cognitive abilities (Dunbar and Shultz 2007). However, there was no relationship between sociability and shoal size discrimination performance among the 10 selected species, which was unexpected. Furthermore, HP species exhibited moderately high sociability with less variation, whereas the LP species showed much higher variation for both sociability variables (i.e., with either extremely high or low sociability). The reason may be that (1) for some species with extremely high sociability, e.g., crucian carp in the present study, fish might seldom leave a group and need not perform shoal size discrimination tasks frequently in the field (Cote et al. 2012; Lucon-Xiccato and Dadda 2017a), whereas (2) for fish with very low sociability, e.g., bitterling in the present study, the lower willingness to stay within a shoal also weakens the numerical ability during the long-term evolution period. Nevertheless, the present study found that all HP species exhibited relatively high sociability with less variation and thus possessed the willingness to join large shoals, while ensuring more exploration behavior, i.e., choosiness, which requires swimming away from the stimulus shoal, i.e., less sociability. In all, we found an interesting correlation between two personality traits and numerical discrimination ability in an ecological-related cognitive task. Further investigation with the aim of understanding correlations between personality and cognitive ability at both inner- and interspecies levels under different ecological contexts (e.g., foraging or mating) might yield interesting results.

### The effect of numerical ratio on shoal presence varied with species

It has been suggested that animals possess two distinct numerical systems operating over different numerical ranges (Feigenson et al. 2004). The ‘object tracking system’ (OTS), which is precise but subject to a set size limit of 4 items (Gómez-Laplaza and Gerlai 2011; Piffer et al. 2012), and the ‘approximate number system’ (ANS) have no upper limit in accordance with Weber's law (Ward and Smuts 2007). Thus, fish species can count on only the ANS system to perform numerical discrimination tasks in a high numerical range (i.e., larger than four), which has been widely accepted. However, whether both the ANS and OTS are present in fish species as they are in human infants (Feigenson and Carey 2005) or whether the ANS is activated over the entire numerical range is quite controversial. It has been suggested that the presence of ratio sensitivity is considered a hallmark of the ANS, whereas ratio insensitivity indicates the recruitment of the OTS in humans (Feigenson et al. 2004). Thus, the present study also aimed to conduct a preliminary investigation on whether fish species rely on different numerical systems to perform shoal size discrimination under small numerical ratio contrasts (below 4 numbers) by comparing the ratio sensitivity of the numerical discrimination performance.

Interestingly, the sensitivity of discrimination performance to the numerical contrast ratio varied with species. In general, the performance (both distance to and percentage time near the shoal) of the five HP species was insensitive to the numerical ratio, whereas the performance of all five LP species varied with the numerical ratio and followed Webber’s law. This suggests that species may rely on different numerical systems in the cognitive task of shoal size discrimination, even within the numerical range of the small number limitation of the DOS (> 4). LP species may use DOS, whereas LP species prefer to use ANS. Because DOS may rely more on numerical information, and ANS prefers to mainly use continual perceptual variables that covary with numbers, the present study might also suggest that species may rely on different quantity traits to select shoals. If so, it is contrary to a previous study that found that most fish species preferred to use continuous quantity traits to select shoals (Agrillo et al. 2008; Pisa and Agrillo 2009; Xiong et al. 2018).

The discrepancy between different studies might be because the mechanism recruited varied under different cognitive tasks with various ecological consequences. This is reasonable because the requirements for speed and accuracy of the numerical ability involved in different ecological tasks, e.g., fighting or escaping from predators, mating and foraging, might be profoundly different or even opposite. In fact, a study on guppies found that quantity discrimination ability varied profoundly between antipredator and foraging contexts (Lucon-Xiccato and Dadda 2017b). Because LP species generally show less shuttling behavior, it is possible that the numerical performance of LP species is more dependent on the first decision of which stimulus shoal the test individual would choose, whereas HP species show more exploration and choice behavior during shoal preference. Thus, ANS might be proper for quick decisions, while OBS requires a more thorough sampling of information, which requires further investigation. Nevertheless, we suggest that selection favors different numerical systems between proactive and reactive species.

## Conclusion

In conclusion, we found evidence that personality covaried with shoal size discrimination ability among freshwater fish species, as bolder species showed higher shoal size discrimination than shyer species probably because those species explored more during the shoal size discrimination task. Furthermore, species with either too high or too low sociability showed low shoal size discrimination. The distinct ratio sensitivity of numerical ability suggests that species with different boldness might recruit different numerical systems, possibly coevolved under certain natural pressures. We also suggest that evolution results in a diversity of both numerical performance and underlying mechanisms between proactive and reactive species for a natural cognitive task in closely related species living in a similar habitat.

## Supplementary Information

Below is the link to the electronic supplementary material.

Supplementary file1 (TIF 34868 KB)

Supplementary file2 (DOCX 18 KB)

## Data Availability

The datasets generated during the current study are available from the corresponding author upon reasonable request.

## References

[CR1] Agrillo C, Bisazza A (2018) Understanding the origin of number sense: a review of fish studies. Philos T Roy Soc B 373:20160511. 10.1098/rstb.2016.0511 10.1098/rstb.2016.0511 PMC5784038 29292358

[CR3] Agrillo C, Dadda M, Serena G, Bisazza A (2008) Do fish count? Spontaneous discrimination of quantity in female mosquitofish. Anim Cogn 11:495–503. 10.1007/s10071-008-0140-9 18247068 10.1007/s10071-008-0140-9

[CR6] Bai Y, Tang ZH, Fu SJ (2019) Numerical ability in fish species: preference between shoals of different sizes varies among singletons, conspecific dyads and heterospecific dyads. Anim Cogn 22(2):133–143. 10.1007/s10071-018-1229-4 30542940 10.1007/s10071-018-1229-4

[CR7] Beckmann C, Biro PA (2013) On the validity of a single (boldness) assay in personality research. Ethology 119(11):937–947. 10.1111/eth.12137

[CR8] Biro PA, Stamps JA (2010) Do consistent individual differences in metabolic rate promote consistent individual differences in behavior? Trends Ecol Evol 25(11):653–659. 10.1016/j.tree.2010.08.003 20832898 10.1016/j.tree.2010.08.003

[CR9] Bisazza A, Gatto E (2021) Continuous versus discrete quantity discrimination in dune snail (Mollusca: Gastropoda) seeking thermal refuges. Sci Rep 11:1–17. 10.1038/s41598-021-82249-6 33580099 10.1038/s41598-021-82249-6 PMC7881015

[CR10] Bisazza A, Santacà M (2022) Zebrafish excel in number discrimination under an operant conditioning paradigm. Anim Cogn 25(4):917–933. 10.1007/s10071-022-01602-y 35179665 10.1007/s10071-022-01602-y PMC9334370

[CR11] Bisazza A, Tagliapietra C, Bertolucci C, Foà A, Agrillo C (2014) Nonvisual numerical discrimination in a blind cavefish (Phreatichthys andruzzii). J Exp Biol 217:1902–1909. 10.1242/jeb.101683 24871921 10.1242/jeb.101683

[CR12] Carazo P, Noble DWA, Chandrasoma D, Whiting MJ (2014) Sex and boldness explain individual differences in spatial learning in a lizard. Proc R Soc B 281:20133275. 10.1098/rspb.2013.3275 24619443 10.1098/rspb.2013.3275 PMC3973267

[CR13] Cattelan S, Lucon-Xiccato T, Pilastro A, Griggio M (2017) Is the mirror test a valid measure of fish sociability? Anim Behav 127:109–116. 10.1016/j.anbehav.2017.03.009

[CR14] Cote J, Fogarty S, Sih A (2012) Individual sociability and choosiness between shoal types. Anim Behav 83:1469–1476. 10.1016/j.anbehav.2012.03.019

[CR15] Daniel DK, Bhat A (2020) Bolder and Brighter? Exploring correlations between personality and cognitive abilities among individuals within a population of wild zebrafsh Danio Rerio. Front Behav Neurosci 14:138. 10.3389/fnbeh.2020.00138 32903664 10.3389/fnbeh.2020.00138 PMC7438763

[CR16] Daniel DK, Bhat A (2023) Correlations begin at home: drivers of co-occurrence patterns in personality and cognitive ability in wild populations of zebrafish. Anim Cogn 26:1381–1394. 10.1007/s10071-023-01787-w 37248284 10.1007/s10071-023-01787-w

[CR17] Dubois F, Binning SA (2022) Predation and parasitism as determinants of animal personalities. J Anim Ecol. 10.1111/1365-2656.13781 35856175 10.1111/1365-2656.13781

[CR18] Dunbar RI, Shultz S (2007) Evolution in the social brain. Science 317:1344–1347. 10.1126/science.1145463 17823343 10.1126/science.1145463

[CR19] Feigenson L, Carey S (2005) On the limits of infants’ quantification of small object arrays. Cognition 97:295–313. 10.1016/j.cognition.2004.09.010 16260263 10.1016/j.cognition.2004.09.010

[CR20] Feigenson L, Dehaene S, Spelke E (2004) Core systems of number. Trends Cogn Sci 8:307–314. 10.1016/j.tics.2004.05.002 15242690 10.1016/j.tics.2004.05.002

[CR21] Ferreira V, Leterrier C, Peuteman B, Valenchon M, Germain K, Brachet M, Leterrier C, Lansade L, Calandreau L, Guesdon V et al (2019) Relationship between ranging behavior and spatial memory of free-range chickens. Behav Proc 166:103888. 10.1016/j.beproc.2019.103888 10.1016/j.beproc.2019.103888 31226335

[CR22] Foster WA, Treherne JE (1981) (1981) Evidence for the dilution effect in the selfish herd from fish predation on a marine insect. Nature 293:466–467. 10.1038/293466a0

[CR23] Giurfa M (2019) Honeybees foraging for numbers. J Comp Physiol A Neuroethol Sens Neural Behav Physiol 205(3):439–450. 10.1007/s00359-019-01344-2 31134327 10.1007/s00359-019-01344-2

[CR24] Gómez-Laplaza LM, Gerlai R (2011) Can angelfish (Pterophyllum scalare) count? Discrimination between different shoal sizes follows Weber’s law. Anim Cogn 14:1–9. 10.1007/s10071-010-0337-6 20607574 10.1007/s10071-010-0337-6

[CR25] Griffin AS, Guez D (2014) Innovation and problem solving: a review of common mechanisms. Behav Proc 109:121–134. 10.1016/j.beproc.2014.08.027 10.1016/j.beproc.2014.08.027 25245306

[CR26] Griffin A, Healy SD, Guillette LM (2015) Cognition and personality: an analysis of an emerging field. Trends Ecol Evol 30(4):207–214. 10.1016/j.tree.2015.01.012 25736691 10.1016/j.tree.2015.01.012

[CR27] Irving E, Brown C (2013) Examining the link between personality and laterality in a feral guppy Poecilia reticulata population. J Fish Biol 83:311–325. 10.1111/jfb.12165 23902308 10.1111/jfb.12165

[CR28] Jolles JW, Boogert NJ, Sridhar VH, Couzin ID, Manica A (2017) Consistent individual differences drive collective behavior and group functioning of schooling fish. Curr Biol 27:2862–2868. 10.1016/j.cub.2017.08.004 28889975 10.1016/j.cub.2017.08.004 PMC5628957

[CR29] Kareklas K, Elwood RW, Holland RA (2017) Personality effects on spatial learning: comparisons between visual conditions in a weakly electric fish. Ethology. 10.1111/eth.12629

[CR30] Killen SS, Fu C, Wu Q, Wang YX, Fu SJ (2016) The relationship between metabolic rate and sociability is altered by food deprivation. Funct Ecol 30(8):1358–1365. 10.1111/1365-2435.12634

[CR31] Krause J, Ruxton GD (2002) Living in groups. Oxford University Press, New York

[CR32] Landeau L, Terborgh J (1986) Oddity and the confusion effect’ in predation. Anim Behav 34:1372–1380. 10.1016/S0003-3472(86)80208-1

[CR33] Liao WB, Jiang Y, Li DY, Jin L, Zhong MJ, Qi Y, Lüpold S, Kotrschal A (2022) Cognition contra camouflage: How the brain mediates predator-driven crypsis evolution. Sci Adv 8(33):eabq878. 10.1126/sciadv.abq1878 10.1126/sciadv.abq1878 PMC9385145 35977010

[CR34] Liu S, Fu SJ (2017) Effects of food availability on metabolism, behaviour, growth and their relationships in a triploid carp. J Exp Biol 220:4711–4719. 10.1242/jeb.167783 29084853 10.1242/jeb.167783

[CR36] Lucon-Xiccato T, Bisazza A (2017) Individual differences in cognition among teleost fishes. Behav Process 141:184–195. 10.1016/j.beproc.2017.01.015 10.1016/j.beproc.2017.01.015 28126389

[CR37] Lucon-Xiccato T, Dadda M (2017a) Personality and cognition: sociability negatively predicts shoal size discrimination performance in guppies. Front Psychol 8:1118. 10.3389/fpsyg.2017.01118 28713317 10.3389/fpsyg.2017.01118 PMC5491838

[CR38] Lucon-Xiccato T, Dadda M (2017b) Individual guppies differ in quantity discrimination performance across antipredator and foraging contexts. Behav Ecol Sociobiol 71:13. 10.1007/s00265-016-2231-y

[CR39] Lucon-Xiccato T, Dadda M, Gatto E, Bisazza A (2017) Development and testing of a rapid method for measuring shoal size discrimination. Anim Cogn 20:149–157. 10.1007/s10071-016-1050-x 27796658 10.1007/s10071-016-1050-x

[CR40] Magurran AE, Seghers BH (1991) Variation in schooling and aggression amongst guppy (Poecilia reticulata) populations in Trinidad. Behaviour 118:214–234. 10.1163/156853991X00292

[CR42] Miller NY, Gerlai R (2008) Oscillations in shoal cohesion in zebrafish (Danio rerio). Behav Brain Res 193:148–151. 10.1016/j.bbr.2008.05.004 18573546 10.1016/j.bbr.2008.05.004 PMC2709827

[CR44] Pérez-Escudero A, Vicente-Page J, Hinz RC, Arganda S, DePolavieja GG (2014) idTracker: tracking individuals in a group by automatic identification of unmarked animals. Nat Method 11:743–748. 10.1038/nmeth.2994 10.1038/nmeth.2994 24880877

[CR45] Piffer L, Agrillo C, Hyde DC (2012) Small and large number discrimination in guppies. Anim Cogn 15:215–221. 10.1007/s10071-011-0447-9 21909934 10.1007/s10071-011-0447-9

[CR46] Pisa PE, Agrillo C (2009) Quantity discrimination in felines: a preliminary investigation of the domestic cat (Felis silvestris catus). J Ethol 27:289–293. 10.1007/s10164-008-0121-0

[CR47] Pulliam HR (1973) On the advantages of flocking. J Theor Biol 38:419–422. 10.1016/0022-5193(73)90184-7 4734745 10.1016/0022-5193(73)90184-7

[CR48] Réale D, Garant D, Humphries MM, Bergeron P, Careau V, Montiglio PO (2010) Personality and the emergence of the pace-of-life syndrome concept at the population level. Proc Royal Soc B 365:4051–4063. 10.1098/rstb.2010.0208 10.1098/rstb.2010.0208 PMC2992747 21078657

[CR49] Reichert MS, Morand-Ferron J, Kulahci IG, Firth JA, Davidson GL, Crofts SJ, Quinn JL (2021) Cognition and covariance in the producer-scrounger game. J Anim Ecol 90(11):2497–2509. 10.1111/1365-2656.13551 34091901 10.1111/1365-2656.13551

[CR52] Schuster AC, Zimmermann U, Hauer C, Foerster K (2017) A behavioral syndrome, but less evidence for a relationship with cognitive traits in a spatial orientation context. Front Zool 14:19. 10.1186/s12983-017-0204-2 28344631 10.1186/s12983-017-0204-2 PMC5364594

[CR53] Shettleworth SJ (2010) Cognition, evolution, and behavior, 2nd edn. Oxford University Press, Oxford, UK

[CR54] Sih A, Bell AM (2008) Insights for Behavioral Ecology from Behavioral Syndromes. Adv Study Behav 38:227–281. 10.1016/S0065-3454(08)00005-3 24991063 10.1016/S0065-3454(08)00005-3 PMC4075144

[CR55] Sih A, Del Giudice M (2012) Linking behavioural syndromes and cognition: a behavioural ecology perspective. Philos Trans r Soc B Biol Sci 367:2762–2772. 10.1098/rstb.2012.0216 10.1098/rstb.2012.0216 PMC3427552 22927575

[CR56] Tang ZH, Fu SJ (2019) Qingbo (Spinibarbus sinensis) personalities and their effect on shoaling behavior. Acta Ethol 22(2):135–144. 10.1111/jfb.14872

[CR57] Thornton A, Isden J, Madden JR (2014) Toward wild psychometrics: linking individual cognitive differences to fitness. Behav Ecol 25:1299–1301. 10.1093/beheco/aru095

[CR58] Trompf L, Brown C (2014) Personality affects learning and trade-offs between private and social information in guppies, Poecilia reticulata. Anim Behav 88:99–106. 10.1016/j.anbehav.2013.11.022

[CR59] Ward C, Smuts BB (2007) Quantity-based judgments in the domestic dog (Canis lupus familiaris). Anim Cogn 10:71–80. 10.1007/s10071-006-0042-7 16941158 10.1007/s10071-006-0042-7

[CR61] White SL, Wagner T, Gowan C, Braithwaite VA (2017) Can personality predict individual differences in brook trout spatial learning ability? Behav Proc 141:220–228. 10.1016/j.beproc.2016.08.009 10.1016/j.beproc.2016.08.009 27567303

[CR62] Wolf M, Weissing FJ (2012) Animal personalities: consequences for ecology and evolution. Trends Ecol Evol 27(8):452–461. 10.1016/j.tree.2012.05.001 22727728 10.1016/j.tree.2012.05.001

[CR63] Wright D, Newton-Youens J, Frommen JG (2023) Four’s a crowd: social preferences for larger groups in golden mantella (*Mantella aurantiaca*) tadpoles. Evol Ecol. 10.1007/s10682-023-10276-y

[CR64] Xiong W, Yi LC, Tang ZH, Zhao X, Fu SJ (2018) Quantity discrimination in fish species: fish use non-numerical continuous quantity traits to select shoals. Anim Cogn 21(6):813–820. 10.1007/s10071-018-1214-y 30242668 10.1007/s10071-018-1214-y

