# Supplementary figures and images for: Personality and cognition: shoal size discrimination performance is related to boldness and sociability among ten freshwater fish species
Source: Anim Cogn. 2024 Mar 2;27(1):6. doi: 10.1007/s10071-024-01837-x (PMC10907435; doi:10.1007/s10071-024-01837-x)

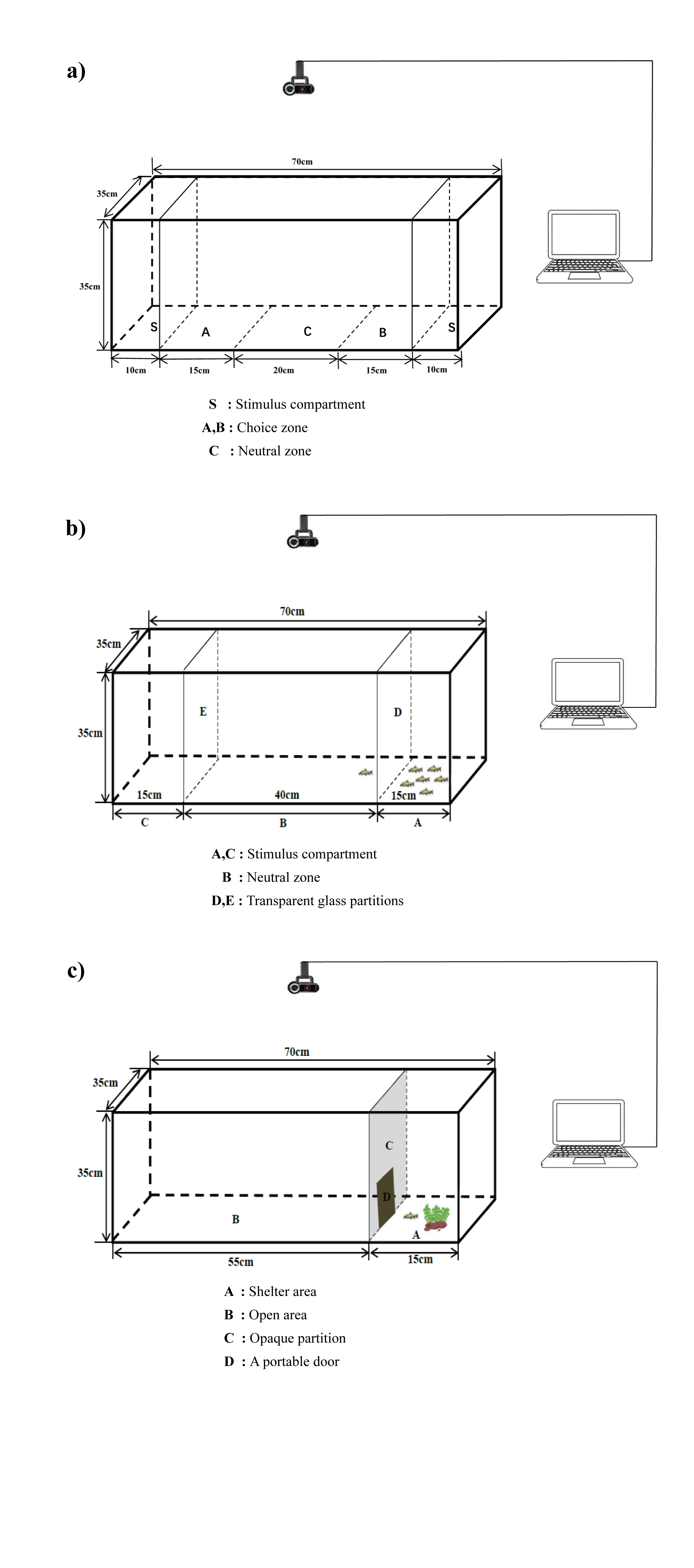

Supplement: Supplementary file 1 — Supplementary file1 (TIF 34868 KB) [file 10071_2024_1837_MOESM1_ESM.tif]
