# Supplementary material for: Personality and cognition: shoal size discrimination performance is related to boldness and sociability among ten freshwater fish species
Source: Anim Cogn. 2024 Mar 2;27(1):6. doi: 10.1007/s10071-024-01837-x (PMC10907435; doi:10.1007/s10071-024-01837-x)
Supplement: Supplementary file 2 — Supplementary file2 (DOCX 18 KB) [file 10071_2024_1837_MOESM2_ESM.docx]

**Supplemental Table 1** The effect of species on the traits of spontaneous movement behavior of fish species during the shoal size discrimination task and personality based on a one-way multivariate analysis of covariance (MANCOVA) (with body mass as the covariate)

|  | Variables | Shuttering frequency | Time in neutral area | Median speed | Time spent moving |
| --- | --- | --- | --- | --- | --- |
| Movement traits during shoal preference | Covariate effect | *F*_1,649_=0.400 | *F*_1,649_=5.623 | *F*_1,649_=2.131 | *F*_1,649_=1.765 |
|  |  | *P*=0.527 | *P*=0.018* | *P*=0.145 | *P*=0.184 |
|  | Species effect | *F*_9,649_=33.14 | *F*_9,649_=13.95 | *F*_9,649_=106.8 | *F*_9,649_=60.37 |
|  |  | *P*<0.001* | *P*<0.001* | *P*<0.001* | *P*<0.001* |
|  | Variables | Inspection frequency | Inspection latency | Time near shoal | Distance to shoal |
| Personality | Covariate effect | *F*_1,184_=0.001 | *F*_1,184_=0.277 | *F*_1,184_=4.133 | *F*_1,184_=0.504 |
|  |  | *P*=0.972 | *P*=0.599 | *P*=0.043* | *P*=0.479 |
|  | Species effect | *F*_9,184_=16.668 | *F*_9,184_=4.935 | *F*_9,184_=2.597 | *F*_9,184_=2.621 |
|  |  | *P*<0.001* | *P*<0.001* | *P*=0.008 | *P*=0.007 |

* Significant at *P* < 0.05

**Supplemental Table 2** The Spearman correlation for variables within the traits of spontaneous movement behavior of fish species during the shoaling preference test and personality among ten species

|  |  |  |  | | Movement trait in shoal preference | |  | | Personality | | | |
| --- | --- | --- | --- | --- | --- | --- | --- | --- | --- | --- | --- | --- |
|  |  |  | Shuttering frequency | Time in neutral area | | Median speed | Time spent moving |  | Inspection frequency | Inspection latency | Time near shoal | Distance to shoal |
| Movement trait in shoal preference | Shuttering frequency |  | */* | *R*=0.915 | | *R*=0.067 | *R*=0.224 |  | *R*=0.661 | *R*=-0.782 | *R*=0.406 | *R*=-0.358 |
|  |  |  |  | *P*<0.001* | | *P*=0.855 | *P*=0.533 |  | *P*=0.038* | *P*=0.008* | *P*=0.244 | *P*=0.310 |
|  | Time in neutral area |  |  | */* | | *R*=0.103 | *R*=0.200 |  | *R*=0.830 | *R*=-0.842 | *R*=0.188 | *R*=-0.188 |
|  |  |  |  |  |  | *P*=0.777 | *P*=0.580 |  | *P*=0.003* | *P*=0.002* | *P*=0.603 | *P*=0.603 |
|  | Median speed |  |  |  | | */* | *R*=0.964 |  | *R*=0.273 | *R*=-0.188 | *R*=0.309 | *R*=-0.309 |
|  |  |  |  |  | |  | *P*<0.001* |  | *P*=0.446 | *P*=0.603 | *P*=0.385 | *P*=0.385 |
|  | Time spent moving |  |  |  | |  | */* |  | *R*=0.321 | *R*=-0.248 | *R*=0.345 | *R*=-0.321 |
|  |  |  |  |  | |  |  |  | *P*=0.365 | *P*=0.489 | *P*=0.328 | *P*=0.365 |
| Personality | Inspection frequency |  |  |  | |  |  |  | */* | *R*=-0.879 | *R*=-0.055 | *R*=0.006 |
|  |  |  |  |  | |  |  |  |  | *P*<0.001* | *P*=0.881 | *P*=0.985 |
|  | Inspection latency |  |  |  | |  |  |  |  | */* | *R*=-0.030 | *R*=0.042 |
|  |  |  |  |  | |  |  |  |  |  | *P*=0.934 | *P*=0.907 |
|  | Time near shoal |  |  |  | |  |  |  |  |  | */* | *R*=-0.879 |
|  |  |  |  |  | |  |  |  |  |  |  | *P*=0.001* |
|  | Distance to shoal |  |  |  | |  |  |  |  |  |  | */* |
|  |  |  |  |  | |  |  |  |  |  |  |  |

* Significant at *P* < 0.05
